# Dramatic improvements in outcome following pancreatoduodenectomy for pancreatic and periampullary cancers

**DOI:** 10.1038/s41416-024-02757-w

**Published:** 2024-06-27

**Authors:** Hui Xu, Michael Bretthauer, Fang Fang, Weimin Ye, Li Yin, Hans-Olov Adami

**Affiliations:** 1 Department of Thoracic Surgery, Fujian Medical University Union Hospital, Fuzhou, China; 2 Department of Medical Epidemiology and Biostatistics, Karolinska Institutet, Stockholm, Sweden; 3 Clinical Effectiveness Research Group, Institute of Health and Society, University of Oslo, Oslo, Norway; 4 Institute of Environmental Medicine, Karolinska Institutet, Stockholm, Sweden; 5 School of Public Health, Fujian Medical University, Fuzhou, China; 6 Department of Epidemiology, Harvard T.H. Chan School of Public Health, Boston, MA USA

**Keywords:** Cancer epidemiology, Pancreatic cancer

## Abstract

**Background:**

Pancreatoduodenectomy is the only cure for cancers of the pancreas and the periampullary region but has considerable operative complications and uncertain prognosis. Our goal was to analyse temporal improvements and provide contemporary population-based benchmarks for outcomes following pancreatoduodenectomy.

**Methods:**

We empanelled a cohort comprising all patients in Sweden with pancreatic or periampullary cancer treated with pancreatoduodenectomy from 1964 to 2016 and achieved complete follow-up through 2016. We analysed postoperative deaths and disease-specific net survival.

**Results:**

We analysed 5923 patients with cancer of the pancreas (3876), duodenum (444), bile duct (504), or duodenal papilla (963) who underwent classic (3332) or modified (1652) Whipple’s procedure or total pancreatectomy (803). Postoperative deaths declined from 17.2% in the 1960s to 1.6% in the contemporary time period (2010–2016). For all four cancer types, median, 1-year and 5-year survival improved substantially over time. Among patients operated between 2010 and 2016, 5-year survival was 29.0% (95% confidence interval (CI): 25.5, 33.0) for pancreatic cancer, 71.2% (95% CI: 62.9, 80.5) for duodenal cancer, 30.8% (95% CI: 23.0, 41.3) for bile duct cancer, and 62.7% (95% CI: 55.5, 70.8) for duodenal papilla cancer.

**Conclusion:**

There is a continuous and substantial improvement in the benefit-harm ratio after pancreatoduodenectomy for cancer.

## Introduction

Pancreatic cancer ranks fourth among cancer-related deaths in the US [1, 2]. Surgical removal in an early stage by pancreatoduodenectomy (so-called Whipple’s procedure) is the only chance for cure for pancreatic and periampullary cancers (cancers of the duodenum or duodenal papilla and the common bile duct). First described in 1935 [3], the Whipple’s procedure has remained the standard treatment for such tumours, either in its classic form [2, 4] or in variations such as pylorus-preserving [5], laparoscopic and robotic surgery [6]. These procedures are extensive and demanding, requiring anastomoses to the gastric remnant, bile duct and pancreatic duct. Postoperative morbidity and mortality have remained deterrent [2] causing large international variations [7] in its use and outcomes [6] although less burdening adverse events have been reported from highly specialised centres [8–10].

With the increasing incidence of pancreatic and periampullary cancers eligible to surgical treatment [1, 2] it is important to assess the benefits as compared to the considerable harms and burdens of the procedure to enable shared decision-making with patients and amongst caregivers. However, no study has defined benchmarks achievable among unselected, contemporary patients in a population-based setting. Further, the impact of radical surgery on long-term survival has not been well documented. Hence, almost a century after it was first described, the use of Whipple’s procedure [3] must be guided by observational data, and a randomised trial comparing radical surgery with palliative treatment is unlikely to see the light of day.

We took advantage of the Swedish Cancer Register with complete nationwide data on incidence and survival of all patients in the country to empanel a large, population-based cohort. We quantified postoperative adverse events (reoperations and deaths) and long-term survival following pancreatoduodenectomy for patients with cancer of the pancreas or periampullary region. We analysed temporal trends in survival during a long period when the procedure has become centralised to fewer centres [10], and new imaging and endoscopic techniques have been introduced for preoperative staging [2].

## Methods

### Study population

Commencing in the 1960s, the National Board of Health and Welfare in Sweden established the Swedish National Patient Register and initiated the systematic acquisition of all inpatient care data within the public healthcare infrastructure of the country. Registration began in defined regions of Sweden (and was therefore strictly population-based) and gradually expanded, culminating in complete coverage by 1987 [11]. This comprehensive database encompasses complete data on patients (e.g., sex, age) and their medical data (e.g., diagnoses, surgical procedures, and dates of admission, discharge, and procedures). The coding of surgical procedures followed the Classification of Operations-sixth edition before 1997 and has since 1997 followed the Classification of Care Measures. Concurrently, the diagnoses in this register have followed, at various periods, the Swedish redivisions of the International Classification of Diseases (ICD) codes (from the 7th revision to the 10th revision).

The Swedish Cancer Register has since its establishment in 1958 covered the entire Swedish population [12]. This register includes information on the specific diagnoses, coded by ICD, and the date of diagnosis. Furthermore, the Swedish Causes of Death Register has been meticulously amassing data since 1952 [13], recording causes as well as date of death for the entire Swedish population, where the causes of death are also coded by ICD. The Migration Register provides information about dates of immigration and emigration since 1947. The Swedish national registration number, uniquely assigned to each resident, serves as the cornerstone for cross-linking data across diverse registers [14].

In this study, we obtained information on sex, date of birth, surgical procedures, and discharge diagnoses from the Patient Register, date of emigration from the Migration Register, and cancer diagnosis from both the Patient Register and the Cancer Register. Our study included all individuals older than 18 years in Sweden who underwent a classic Whipple’s procedure, a modified Whipple’s procedure, or a total pancreatectomy between January 1964 and December 2016. No linked register data were available from 2017 and onwards.

We focused on a primary diagnosis of four specific types of cancer: pancreatic cancer, duodenal cancer, bile duct cancer, and duodenal papilla cancer. Individuals with a diagnosis of other diseases or secondary cancers, as well as individuals with no underlying diagnosis as an indication for the procedures, were excluded. Because pancreatic neuroendocrine tumours exhibit significantly different clinical characteristics than the predominant pancreatic ductal adenocarcinomas, we also excluded these patients from our study. We further excluded individuals with a death record but with a missing date of death, thus precluding survival analysis. We followed the remaining individuals from the date of surgery until death, emigration, or end of follow-up (December 31, 2016), whichever came first. A detailed description of the stepwise definition of our analytic cohort is provided in Supplementary Fig. S1.

### Surgical procedures and underlying diagnoses

The sixth edition of the Classification of Operations was used in the Patient Register from 1963 until 1996, employing a four-digit procedure coding system. We accordingly used the codes 5512, 5513, and 5516 to identify classic Whipple’s procedure, modified Whipple’s procedure, and total pancreatectomy, respectively, before 1997. The Classification of Surgical Measures and Medical Measures with a dual code structure has been used in the Patient Register since 1997. Accordingly, we used the codes JLC30, JLC40, and JLC20 to identify the above three procedures, since 1997 onward. We could, however, not identify pylorus-preserving procedures, which accounted for 23.3% of the procedures during 2010–2017 [10]. For further details, please refer to Supplementary Table S1. Details on the ICD codes used to identify the different cancer types are shown in Supplementary Table S2.

### Outcomes

We analysed death with pancreatic cancer, duodenal cancer, bile duct cancer, or duodenal papilla cancer as the underlying cause as the primary outcome of interest, according to the Causes of Death Register, during the follow-up. Patients with other causes of death were censored at the date of death. We also studied reoperation, defined as any abdominal surgery performed after pancreaticoduodenectomy during the same hospitalisation, and postoperative mortality, as death within 30 days after pancreaticoduodenectomy. Detailed information on codes used to identify disease-specific deaths is provided in Supplementary Table S2.

### Statistical analyses

We described patient characteristics, where continuous variables were presented as means ( ± SD) and categorical variables as percentages, and we calculated the percentage of reoperation and postoperative mortality between different groups. We analysed disease-specific survival as a measure of net survival [15]. Median survival time in months and disease specific 1- and 5-year survival in proportion with 95% confidence intervals (CI) for each group was calculated using the Kaplan–Meier method, and into group differences were assessed using the log-rank test.

We employed multivariable logistic regression to compare the rates of reoperation and postoperative mortality between different groups. Because the hazard ratio (HR) of disease-specific mortality varied with time, we employed kernel smoothing of the Nelson-Aalen estimator to obtain time-varying smooth hazard estimates from a Cox model. We then calculated the restricted mean survival ratio (RMST) with adjustment for sex, age, calendar time, underlying diagnosis and type of surgery to assess the differences in disease-specific mortality between groups. A *P* value less than 0.05 was considered statistically significant. Data management was performed using SAS software version 9.4 (SAS Institute), and all statistical analyses were carried out using R software version 4.2.3 (R Foundation for Statistical Computing).

## Results

### Patient characteristics

Our study cohort consisted of 5787 individuals who had undergone pancreaticoduodenectomy for one of the four cancer types from 1964 to 2016 in Sweden. Of these, 3876 had pancreatic cancer, 444 had duodenal cancer, 504 had cancer in the common bile duct, and 963 had cancer of the duodenal papilla. The mean age was 65.1 years, and 47% of patients were women. The total number of procedures increased from 29 in the 1960s to 1801 during 2010–2016. Classic Whipple’s procedure accounted for 3332 of the procedures, while 1652 patients were operated using the modified Whipple’s procedure and 803 with total pancreatectomy (Table 1).

**Table 1:** Characteristics of the study cohort, number (%) of reoperations and postoperative deaths.

Characteristics	Entire cohort	Individuals with reoperation	Individuals with postoperative mortality
Sex—no. (%)			
Women	2728 (47.1)	133 (4.9)	82 (3.0)
Men	3059 (52.9)	215 (7.0)	138 (4.5)
Total	5787	348 (6.0)	220 (3.8)
Age at surgery (mean age ± SD)—yr			
Women	65.5 ± 9.7	67.0 ± 9.1	68.3 ± 7.7
Men	64.7 ± 9.6	65.3 ± 9.4	66.4 ± 7.9
Total	65.1 ± 9.6	66.0 ± 9.3	67.1 ± 7.9
Age at surgery, yr—no. (%)			
<50	386 (6.7)	21 (5.4)	5 (1.3)
50–59	1048 (18.1)	45 (4.3)	30 (2.9)
60–69	2263 (39.1)	145 (6.4)	92 (4.1)
≥70	2090 (36.1)	137 (6.6)	93 (4.5)
Year of surgery—no. (%)			
1964–1969	29 (0.5)	–	5 (17.2)
1970–1979	338 (5.8)	–	60 (17.8)
1980–1989	854 (14.8)	–	56 (6.6)
1990–1999	1067 (18.4)	58 (5.4)	30 (2.8)
2000–2009	1698 (29.3)	134 (7.9)	41 (2.4)
2010–2016	1801 (31.1)	110 (6.1)	28 (1.6)
Underlying diagnosis—no. (%)^a^			
Pancreatic cancer	3876 (67.0)	206 (5.3)	151 (3.9)
Duodenal cancer	444 (7.7)	39 (8.8)	13 (2.9)
Bile duct cancer	504 (8.7)	37 (7.3)	17 (3.4)
Duodenal papilla cancer	963 (16.6)	66 (6.9)	39 (4.1)
Types of operation—no. (%)^b^			
Classic Whipple’s procedure	3332 (57.6)	210 (6.3)	117 (3.5)
Modified Whipple’s procedure	1652 (28.5)	94 (5.7)	80 (4.8)
Total pancreatectomy	803 (13.9)	44 (5.5)	23 (2.9)

^a^Defined according to ICD-7 to ICD-10 codes for different time periods (supplemental content contains the specific ICD codes).

^b^Defined according to codes of classification of healthcare measures (KVÅ) for different time periods (supplemental content contains the specific KVÅ codes).

### Adverse events

Data on reoperation has only been available since 1990. The proportion of patients undergoing any reoperation was in the range of 5 to 10% during all time periods after 1990. We found no obvious difference between calendar periods for the four cancer types or surgical procedures. In contrast, postoperative mortality decreased monotonically and drastically from 17.2% in the 1960s to 1.6% during the most recent time period with no appreciable difference between the four cancer types and the three surgical procedures but an increasing trend with higher age at pancreatoduodenectomy (Table 1).

### Disease-specific survival

**Fig. 1:**
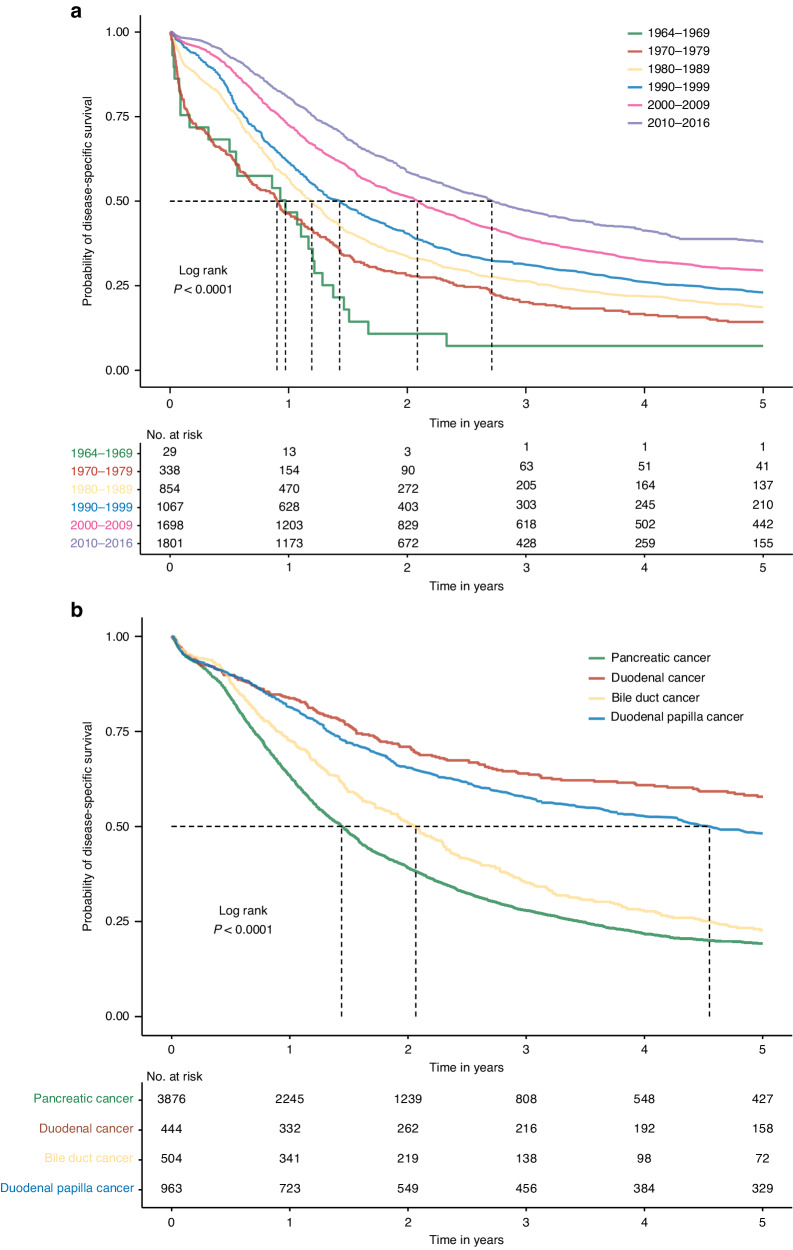
Disease-specific survival. **a** Disease-specific survival for patients who underwent classic Whipple’s procedure, modified Whipple’s procedure, or total pancreatectomy between 1964 and 2016, by year of operation. **b** Disease-specific survival for patients who underwent classic Whipple’s procedure, modified Whipple’s procedure, or total pancreatectomy, by cancer type.

For all four cancer types combined, the median survival increased from 11.7 months in the 1960s to 32.7 months during 2010–2016 (Table 2). 1-year survival increased from 46.7 to 80.5%, and 5-year survival increased from 7.2 to 37.9%. The successive improvement in survival is illustrated in Fig. 1a and Supplementary Fig. S2. We also found significant differences between the four cancer types. Overall, the median survival ranged from 17.4 months in pancreatic cancer, 24.9 months in bile duct cancer, to 54.9 months in duodenal papilla cancer. The 1-year survival ranged from 63.2 to 83.7%, and 5-year survival from 19.2 to 57.8%. These differences in survival are shown in Fig. 1b and Supplementary Table 2.

**Table 2:** Median, 1-year and 5-year disease-specific survival.

Characteristics	Median survival in months	Survival in proportion (95% CI)	
		1-year	5-year
Sex			
Women	22.0	69.7 (68.0–71.5)	26.8 (25.0–28.7)
Men	21.3	67.6 (65.9–69.3)	28.1 (26.4–30.0)
Total	21.7	68.6 (67.4–69.8)	27.5 (26.2–28.8)
Age at surgery, yr			
<50	24.1	72.0 (67.6–76.7)	33.9 (29.2–39.4)
50–59	22.0	68.6 (65.8–71.5)	29.0 (26.2–32.1)
60–69	22.5	69.6 (67.6–71.5)	27.3 (25.4–29.4)
≥70	19.9	67.0 (64.9–69.1)	25.5 (23.4–27.8)
Year of surgery			
1964–1969	11.7	46.7 (31.4– 69.4)	7.2 (1.9–27.3)
1970–1979	10.9	46.4 (41.3–52.0)	14.3 (10.9–18.7)
1980–1989	14.4	56.5 (53.2–59.9)	18.7 (16.1–21.6)
1990–1999	17.3	61.4 (58.5–64.4)	23.0 (20.5–25.8)
2000–2009	25.2	72.4 (70.3–74.6)	29.5 (27.4–31.8)
2010–2016	32.7	80.5 (78.6–82.5)	37.9 (34.9–41.1)
Underlying diagnosis^a^			
Pancreatic cancer			
1964–2016	17.4	63.2 (61.7–64.8)	19.2 (17.8–20.7)
2010–2016	26.7	78.0 (75.5–80.5)	29.0 (25.5–33.0)
Duodenal cancer			
1964–2016	^c^	83.7 (80.2–87.3)	57.8 (53.0–63.1)
2010–2016	^c^	91.5 (87.1–96.0)	71.2 (62.9–80.5)
Bile duct cancer			
1964–2016	24.9	72.6 (68.8–76.7)	22.7 (18.9–27.2)
2010–2016	29.4	82.6 (77.0–88.6)	30.8 (23.0–41.3)
Duodenal papilla cancer			
1964–2016	54.9	81.3 (78.8–83.8)	48.2 (44.9–51.7)
2010–2016	^c^	84.3 (79.5–89.5)	62.7 (55.5–70.8)
Types of operation^b^			
Classic Whipple’s procedure	22.9	70.9 (69.3–72.5)	28.8 (27.1–30.6)
Modified Whipple’s procedure	20.8	67.1 (64.9–69.5)	26.0 (23.8–28.5)
Total pancreatectomy	18.0	62.2 (58.9– 65.8)	25.4 (22.3–28.9)

^a^Defined according to ICD-7 to ICD-10 codes for different time periods (supplemental content contains the specific ICD codes).

^b^Defined according to codes of classification of healthcare measures (KVÅ) for different time periods (supplemental content contains the specific KVÅ codes).

^c^Impossible to estimate the median survival due to a high proportion of individuals remaining alive at the end of the follow-up.

Survival deteriorated with increasing age. Median survival decreased from 24.1 months among patients younger than 50 years to 19.9 months among those who were 70 years or older (Table 2). Corresponding 1-year survival decreased from 72.0 to 67.0% and 5-year survival from 33.9 to 25.5%.

Because results in patients operated most recently may be decisive for contemporary management, we calculated the median and 5-year survival among patients operated between 2010 and 2016. The 5-year survival ranged from 29.0% in patients with pancreatic cancer to 71.2% in those with duodenal cancer (Table 2). We also plotted annual hazards to illustrate how excess deaths from the underlying malignancy evolved during follow-up (Supplementary Fig. S3), which demonstrated that, in all categories, the hazard peaked about one year after operation and approached zero within five years as an indicator of cure.

### Comparison of adverse events and disease-specific survival

In the multivariable analysis (Table 3), we found a 95% reduction in postoperative mortality from the 1960s to the most recent period. The risk of reoperation and postoperative mortality was more than 40% higher in men than in women. Whilst the risk of reoperation was largely unrelated to age, the risk of postoperative mortality increased markedly with age and was more than sevenfold higher in patients who were 70 years or older compared with those who were less than 50 years. Patients who underwent reoperation experienced a threefold increased risk of postoperative mortality (Table 3).

**Table 3:** Reoperation, postoperative death, and disease-specific survival in relation to patient characteristics, after multivariable adjustment.

Characteristics	Reoperation	Postoperative mortality	RMST	
	Relative risk (95% CI)^c^		Difference (95% CI)	Ratio (95% CI)^c^
Sex				
Women	1.0	1.0	1.0	1.0
Men	1.47 (1.16–1.87)	1.60 (1.20–2.14)	−0.05 (−0.14 to 0.05)	0.98 (0.94–1.02)
Age at surgery, yr				
<50	1.0	1.0	1.0	1.0
50–59	0.80 (0.44–1.54)	2.70 (1.10–8.10)	−0.24 (−0.49 to 0.01)	0.92 (0.83–1.00)
60–69	1.41 (0.83–2.54)	4.30 (1.87–12.48)	−0.26 (−0.49 to −0.03)	0.91 (0.84–0.99)
≥70	1.34 (0.79–2.42)	7.59 (3.27–22.19)	−0.70 (−0.93 to −0.47)	0.76 (0.69–0.83)
* P* for trend	0.04	<0.001	<0.001	<0.001
Year of surgery				
1964–1969	–	1.0	1.0	1.0
1970–1979	–	1.19 (0.46–3.71)	0.41 (−0.14 to 0.96)	1.47 (0.91–2.36)
1980–1989	–	0.32 (0.12–0.98)	0.81 (0.29–1.33)	1.80 (1.13–2.86)
1990–1999	1.0	0.10 (0.04–0.33)	1.20 (0.67–1.73)	2.13 (1.34–3.39)
2000–2009	1.51 (1.03–2.23)	0.08 (0.03–0.25)	1.65 (1.14–2.16)	2.53 (1.60–4.01)
2010–2016	1.09 (0.74–1.61)	0.05 (0.02–0.15)	1.23 (0.72–1.73)	2.15 (1.36–3.39)
* P* for trend	0.62	<0.001	<0.001	<0.001
Underlying diagnosis^a^				
Pancreatic cancer	1.0	1.0	1.0	1.0
Duodenal cancer	1.78 (1.21–2.55)	0.91 (0.48–1.59)	1.44 (1.24–1.65)	1.69 (1.59–1.80)
Bile duct cancer	1.39 (0.93–2.02)	1.02 (0.58–1.68)	0.37 (0.18–0.57)	1.18 (1.09–1.29)
Duodenal papilla cancer	1.39 (1.00–1.90)	0.83 (0.57–1.20)	1.41 (1.26–1.56)	1.70 (1.62–1.79)
Types of operation^b^				
Classic Whipple’s procedure	1.0	1.0	1.0	1.0
Modified Whipple’s procedure	1.03 (0.76–1.37)	0.70 (0.50–0.97)	0.10 (−0.02 to 0.22)	1.05 (0.99–1.11)
Total pancreatectomy	1.00 (0.65–1.50)	0.87 (0.49–1.47)	0.01 (−0.18 to 0.20)	1.01 (0.93–1.10)
Reoperation				
No	–	1.0	1.0	1.0
Yes	–	3.41 (2.19–5.16)	−0.36 (−0.58 to −0.14)	0.86 (0.78–0.96)

^a^Defined according to ICD-7 to ICD-10 codes for different time periods (supplemental content contains the specific ICD codes).

^b^Defined according to codes of classification of healthcare measures (KVÅ) for different time periods (supplemental content contains the specific KVÅ codes).

^c^Relative risk was calculated based on logistic regression whereas ratio was calculated as restricted mean survival ratio (RMST), after adjustment for sex, age, calendar period, underlying diagnosis, and type of surgery.

**Fig. 2:**
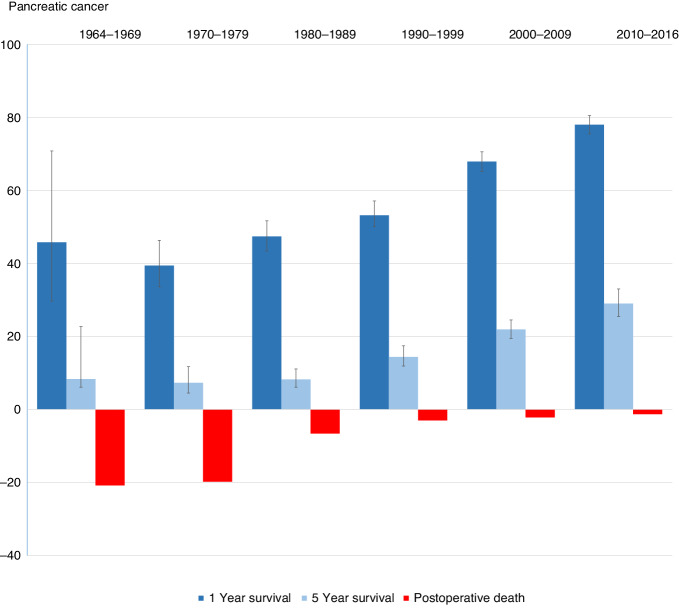
Postoperative death, 1-year survival and 5-year survival among individuals with the Whipple procedure for pancreatic cancer from 1970 to 2016 in Sweden. The 1-year and 5-year survival rates were calculated using the Kaplan–Meier method, with error bars indicating the 95% confidence intervals (CI). Adverse events were reported as percentages of postoperative deaths.

Multivariable analyses based on RMST confirmed findings from univariate analyses (Table 3). The RMST increased significantly and monotonically over calendar time and decreased with increasing age. Compared with pancreatic cancer, the prognostic outlook was significantly better for the other three malignancies and largely identical for cancers of the duodenum and duodenal papilla. Reoperation was associated with a lower survival. Compared with patients operated in the 1960s, those operated on recently exhibited a significantly increased ratio for RMST at 2.15 (95% CI: 1.36, 3.39). In addition, long-term prognosis deteriorated monotonically with increasing age, the RMST ratio for patients aged 70 years or older was 0.76 (95% CI: 0.69, 0.83) compared to those younger than 50 years. In contrast, we found no significant association between sex or type of operation and disease-specific survival. Given that pancreatic cancer constituted almost 70% of all four cancer types, we illustrated adverse events and survival for pancreatic cancer in Fig. 2, separately.

## Discussion

Our most salient findings were the dramatic reductions in postoperative deaths and the improvements in prognosis over time and the substantial long-term cure among the most recently operated patients. We also found large differences in the long-term outcomes between the four malignancies and worse short- and long-term outcomes with increasing age. In contrast, we found no appreciable differences in long-term outcome between classic and modified Whipple’s procedure and similar results following total pancreatectomy.

Strengths of our study include its large size, the long period of enrollment, complete follow-up and access to high-quality, virtually complete registers covering the entire Swedish population. Because Sweden has a public healthcare system with almost no private inpatient care during the period of our study, the results are strictly population-based. We were also able to analyse disease-specific (net) survival thereby eliminating the confounding influence of competing causes of death between age-groups. This measure should allow valid comparisons with outcomes in countries where patients operated for pancreatic and periampullary cancer may have a different burden of comorbidities [10].

Our study also has potentially important limitations. One is the lack of data on tumour stage in the Swedish Cancer Register. We were therefore unable to investigate whether temporal improvements in survival also took place within strata of the tumour stage. Such analyses may, however, not be valid because tumour staging has likely become more accurate over time which introduces spurious improvements by tumour stage [11]. Still in 2014–2017 the majority (66.6%) of operated patients had locally advanced (T3) tumours and 70.6% had positive regional lymph nodes [10]. We further lacked information on adjuvant radiation- and chemotherapy which may modestly improve overall survival [2]. However, in 2010–2017 only 3.4% of Swedish patients received preoperative chemotherapy [10]. Nevertheless, the median survival among Swedish patients with pancreatic cancer operated between 2010 and 2016 was 26.7 months (Table 2) compared with 20 to 22 months in randomised trials of adjuvant treatment [2].

We lacked detailed information about surgical techniques. Pylorus-preserving operation, described already in the 1940s [5], could not be distinguished from classical Whipple’s procedure, nor the various strategies to create continuity to the small intestine from the gastric remnant, bile duct and pancreatic duct. Unfortunately, surgical details are lacking also in emerging registries of pancreatic surgery in the US and Europe [10, 12]. Finally, pancreatoduodenectomy has been gradually centralised to a limited number of centres in Sweden reaching six (in a population of about 10 million) in 2017 [10]. Although we were interested in analysing whether causal associations—as has been proposed for pancreatoduodenectomy [13] and other surgical procedures [14, 15]—exist between volume and cancer outcomes the relevant information could not be retrieved from register data.

We consider the interpretation of our findings rather straightforward. Hence, increasing specialisation and centralisation to a few high-volume centres has drastically reduced risk of postoperative death. That the surgical procedure has become more curative in recent years than in the past seems unlikely. More plausibly, preoperative imaging techniques and endoscopic examination with high sensitivity [2] have allowed patients with potentially curable cancer to be more reliably identified preoperatively. In particular imaging allows identification of distant metastases and tumours that are unresectable due to involvement of the superior mesenteric artery or coeliac axis. Because we found no evidence that temporal improvement in survival has leveled (Supplementary Fig. S2) we might expect even better prospects for cure among contemporary and future patients undergoing pancreatoduodenectomy. As soon as record linkage data beyond 2016 becomes available, updated analyses are justified.

Because patients with any of the four different malignancies we analysed have presumably undergone largely the same preoperative staging procedure, differences in prognosis may not be substantially confounded by tumour stage; they more likely reflect differences in biologic features. In contrast, comparisons between classic and modified Whipple’s procedure might be confounded by differences in the extent of disease and tumour stage. The largely identical outcomes therefore need cautious interpretation.

Comparison of our results with those in the published literature is not straightforward. The strictly population-based design should make our results widely generalisable. In contrast, existing evidence is largely based on studies reporting postoperative outcomes but no survival data [8, 10, 16, 17], single-institution cohorts [18], patients from highly specialised referral centres [9] or even cohorts operated by a single surgeon [8]. Although benchmarks for short-term outcomes after Whipple’s procedure have been recently proposed [9], they were based on selected low-risk patients with no significant comorbidities treated at expert centres and were limited to short-term morbidity with no data on survival. In the US, only 18% of all patients undergoing Whipple’s procedure were utilised to define these benchmarks [9]. We suggest, therefore, that our results provide benchmarks to be achieved in every hospital that provides surgical treatment for pancreatic and periampullary cancer. Adverse events and long-term survival would then be the systematic outcome measures that define competent providers [19].

## Conclusions

We conclude that pancreatoduodenectomy deserve to be part of standard management for cancer of the pancreas and periampullary region. Further, that optimal preoperative staging may entail that an even larger proportion of operated patients achieve long-term cure. Finally, although data-rich registries are now established in the US and Europe [10], randomised trials are needed to clarify if the different surgical techniques influence the risk of postoperative complications, quality of life, or long-term survival.

### Supplementary information

Supplementary material

## Data Availability

Because the analysis was based solely on population-based registers in Sweden, the investigators are not in the position of owning any data that can be shared.

## Code availability

Please contact the corresponding author for access to codes used to generate the results of the present study.
