# Supplementary material for: Dramatic improvements in outcome following pancreatoduodenectomy for pancreatic and periampullary cancers
Source: Br J Cancer. 2024 Jun 27;131(4):747–54. doi: 10.1038/s41416-024-02757-w (PMC11333598; doi:10.1038/s41416-024-02757-w)
Supplement: Supplementary file 1 — Supplementary material [file 41416_2024_2757_MOESM1_ESM.docx]

**Supplementary material**

|  |  | Page |
| --- | --- | --- |
| 1 | Figure S1 | 2 |
| 2 | Table S1 | 3 |
| 3 | Table S2 | 4 |
| 4 | Figure S2 | 5 |
| 5 | Figure S3 | 6 |

**Figure S1.** Flow chart for recruitment of the study population.

*Inclusion criteria:

1. Participants must be 18 years of age or older.
2. Study period: January 1964 to December 2016.

**Table S1.** Codes used for surgical procedures before and after 1997 in Sweden.

| Operation | Before 1997 | After 1997 |
| --- | --- | --- |
| Classic Whipple procedure | 5512 | JLC30 |
| Modified Whipple procedure | 5513 | JLC40 |
| Total pancreatectomy | 5516 | JLC20 |
| Reoperation | - | JW- |
|  | - | JAK00 |
|  | - | UJD02 |

**Table S2.** Codes used to identify the four cancer types.

| Cancer | ICD-7 | ICD-8 | ICD-9 | ICD-10 |
| --- | --- | --- | --- | --- |
| Pancreatic cancer | 157 | 157.01 | 157A | C25.0 |
|  | 1955 | 157.87 | 157B | C25.1 |
|  |  | 157.88 | 157C | C25.2 |
|  |  | 157.99 | 157D | C25.3 |
|  |  |  | 157E | C25.4 |
|  |  |  | 157W | C25.7 |
|  |  |  | 157X | C25.8 |
|  |  |  |  | C25.9 |
| Duodenal cancer | 152.0 | 152.01 | 152A | C17.0 |
| Bile duct cancer | 155.1 | 156.11 | 156B | C24.0 |
| Duodenal papilla cancer* | 155.1 | 156.21 | 156C | C24.1 |

*As the same code was used for choledochus cancer and papilla Vateri cancer in ICD-7, both these two cancers are identified using the data from variable ICD-8.

**Figure S2. Disease-specific survival during the first one- or five-years following Whipple’s procedure.**

The lines illustrate the changes in 1-year and 5-year survival probability from 1964 to 2016, with survival probability obtained from Kaplan-Meier (KM) curve analysis.


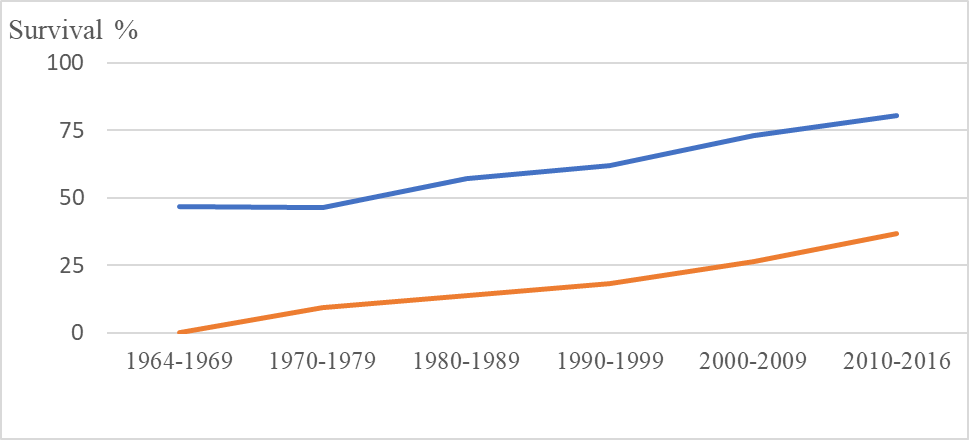


1 year

5 years

**Figure S3. Cumulative hazard estimates in different groups**

Kernel smoothing of the Nelson-Aalen estimator is employed to display hazard estimates from a Cox model, revealing how the hazard varies with follow-up time across different groups.
